# Achieving the end game: employing “vaccine diplomacy” to eradicate polio in Pakistan

**DOI:** 10.1186/s12889-019-6393-1

**Published:** 2019-01-17

**Authors:** Shahella Idrees Shakeel, Matthew Brown, Shakeel Sethi, Tim K. Mackey

**Affiliations:** 1 Joint Master’s Program in Health Policy and Law, University of California, San Diego School of Medicine – California Western School of Law, San Diego, CA USA; 2 National Institutes of Health, National Cancer Institute, U.S. Embassy, China Office, Beijing, China; 3 Combined Military Hospital, Rawalpindi, Pakistan; 4 Department of Anesthesiology and Division of Infectious Diseases and Global Public Health, University of California, San Diego School of Medicine, San Diego, CA USA; 5 Global Health Policy Institute, 8950 Villa La Jolla Drive, A124, La Jolla, San Diego, CA 92037 USA

**Keywords:** Wild polio virus (WPV), Circulatory vaccine derived polio virus (cVDPV), Acute flaccid paralysis (AFP), Vaccine diplomacy, Polio eradication, Health diplomacy, Global health diplomacy (GHD)

## Abstract

**Background:**

On April 28, 2014, the World Health Organization (WHO) declared polio a “Public Health Emergency of International Concern” (PHIC) under the authority of the International Health Regulations. Although polio has been eradicated from nearly every nation on earth, Pakistan is one of three countries where wild polio and vaccine-derived polio strains remain, thwarting global eradication efforts.

**Aims:**

Polio eradication progress is complicated by security and conflict issues at the border area between Pakistan and Afghanistan. In addition to security issues, other critical challenges, such as maintaining cold supply chain for vaccines, active and sentinel surveillance, false beliefs about vaccines, distrust of healthcare workers, and accessibility to conflict areas due to terrorist activities, all play a role in the continued persistence of Polio. In response to these challenges, we assess the local and international policy environment and its impact on polio eradication in Pakistan.

**Findings:**

Based on our analysis of existing barriers and challenges associated with polio eradication in Pakistan, this study discusses why employing “vaccine diplomacy” represents a key policy and advocacy strategic approach to achieve the overall end game of polio eradication. Specifically, we identify a set of concrete public health, international development, and diplomatic and policy recommendations that can act synergistically under the umbrella of health and vaccine diplomacy to finally put an end to polio.

## Background

In April 2014, an emergency committee organized under the International Health Regulations (IHR) of the World Health Organization (WHO), declared the spread of wild poliovirus a “Public Health Emergency of International Concern” [1]. The PHEIC was issued primarily to catalyze political advocacy around polio and to use WHO’s IHR normative powers to activate temporary recommendations to prevent further spread and interrupt wild poliovirus transmission.

Polio (poliomyelitis) is a viral infection with three serotypes transmitted along the fecal–oral route. Although polio is preventable, once contracted it can lead to mortality or significant morbidity due to progression to acute-flaccid paralysis [2]. Two vaccines are effective for prevention, one is a live attenuated oral polio virus vaccine (OPV) and the other is inactivated injectable polio virus (IPV) [3]. Polio has been eradicated in all but three countries globally (Pakistan, Afghanistan, and Nigeria), though ongoing threat of international spread was highlighted in the 2014 PHEIC declaration, which covered affected State Parties of Pakistan, Afghanistan, Nigeria, Syria, Cameroon, Equatorial Guinea, Ethiopia, Israel, and Somalia [4].

**Fig. 1:**
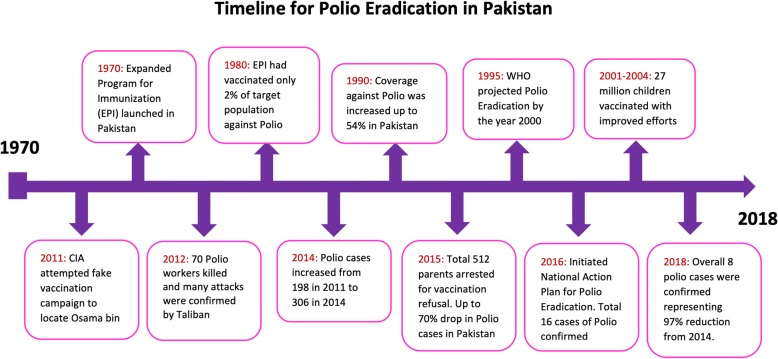
Polio eradication summary timeline (uploaded separately)

Pakistan, a country specifically impacted by ongoing polio transmission, has been at the center of increased international efforts to eradicate polio (see a summary timeline in Fig. 1. During the 14th WHO IHR Emergency Committee, member states specifically noted the large decline in polio cases since 2014 in countries such as Pakistan despite facing challenging circumstances in border areas [5]. Despite this progress, Pakistan and Afghanistan continue to report cases of wild polio virus type-1 (WPV1), one of the primary triggers for the issuance of the PHEIC [6]. The committee also noted the presence of positive environmental samples for wild polio virus (WPV) and circulatory vaccine derived polio virus (cVDPV) in both countries, indicating that vaccine and prevention efforts require constant vigilance.

Importantly, the domestic and international policy environment can increase risks of polio transmission at the micro environmental, community, border, and public health level. Focusing on Pakistan, one of the last strongholds of polio, this article examines characteristics of the policy and governance environment that continue to impede efforts to end this age-old global health threat. Based on our assessment of these challenges, we outline why “vaccine diplomacy” is a useful strategic approach to achieve the polio end game and suggest a set of policy recommendations to achieve this goal.

## Polio in Pakistan: An elusive road to eradication

### Characteristics of polio in Pakistan

Pakistan is among three countries—Pakistan, Afghanistan and Nigeria—that were subject to 2014 IHR temporary travel recommendations as they harbored the potential of spreading polio internationally due to presence of infected individuals with one of the three polio serotypes: WPV1, cVDPV1 or cVDPV3. Concomitantly, the international prevalence of wild poliovirus has dramatically decreased—with no global spread in 2015 and 2016, except between Afghanistan and Pakistan [4]. In 2015, four out of six WHO regions were declared polio-free, while Pakistan and Afghanistan remained an endemic reservoir of wild polio virus [6].

Pakistan is also one of the most populous countries in WHO’s Eastern Mediterranean Region—approximately 207 million people, which includes 24.7 million children under 5 years old and 66.2 million under 15 years old, making child immunization a key challenge in overcoming polio [7]. Pakistan is divided into four provinces (e.g., Sindh, Punjab, Khyber Pakhtunkhwa [KP] and Baluchistan), the Islamabad Capital Area, the federally administered tribal areas (FATA), and two administrative areas (Gilgit-Baltistan and Azad Jammu Kashmir) [8]. Like the rest of the world, Pakistan has successfully eradicated smallpox, though polio remains circulating.

Based on official data shared by the Pakistani government, more than 40% of the 116 WPV cases that occurred during 2014 were a result of vaccination refusal by families [9]. Most polio cases occurred in FATA (an area neighboring Khyber Pakhtunkhwa (KPA) and in the Baluchistan border area with Afghanistan. Combined political instability, security hazards, lower literacy levels, and local religious beliefs present a complex picture of the multitude of challenges associated with eradicating this infectious disease in an area sensitive to social and politically-related risk factors [10, 11].

In 2016–2017, the Pakistani Ministry of Health, Regulations and Coordination, the National Provincial and FATA Emergency Operations Centers, and donors and technical partners such as the WHO, the Food and Agriculture Organization (FAO), U.S. Centers for Disease Control and Prevention (CDC), United States Agency for International Development (USAID), World Bank, United Kingdom Department for International Development (DFID), Public Health England (PHE), and Japan International Cooperation agency (JICA), jointly developed the National Emergency Action Plan (NEAP) [12]. The Plan’s strategic objectives are to eradicate wild polio transmission and sustain disease interruption through increased population immunity via open communication, dedicated efforts, and multi-stakeholder cooperation [13].

However, efforts to implement the NEAP have been impacted by military missions in conflicted areas like North Waziristan, where wild polio cases increased due to immunization bans and immunity gaps [14]. In addition to internal and border conflicts, external military and intelligence operations have created distrust among Pakistanis regarding vaccination campaigns. In 2011, the U.S. Central Intelligence Agency (CIA) funded a fake Hepatitis B vaccination program in the KPA province to garner intelligence on the Saudi terrorist Osama bin Laden [15, 16]. This well documented intelligence operation posing as a public health program fostered local people’s distrust of the public health sector, especially in the context of vaccination programs in KPA. This distrust has persisted among communities living along the border between Afghanistan and Pakistan, where people believe that a polio vaccine could make them sterile and has also spread to other provinces like Baluchistan (Quetta).

Despite these challenges, there has been progress in the fight against polio in Pakistan; based on global surveillance data from March 9, 2016, only 12 cases of wild polio were reported in Pakistan [17]. Further illustrating this progress, there has been an 82% decline of WPV cases (from 306 confirmed in 2014 to only 54 cases in 2015, with only 13 cases of wild polio confirmed in 2016 [18]). However, paralytic polio cases due to cVDPV remained as high as 32 in 2015 [18]. The country also reported a decline in the number of confirmed environmental specimens of wild polio from 35% in 2014 to 10% in 2016 [4]. Also, Pakistan has reduced circulatory genetic clusters of polio from 16 in 2014 to eight in 2015 [19].

Successful declines likely reflect efforts of increased national polio vaccination efforts, with approximately 38.8 million children vaccinated during the month of November in 2017 alone and 1.26 million children vaccinated at 373 permanent transit points [20, 21]. These transit points (e.g. bus stops, railway stations and highways) are setup all over the country and at district borders, in an attempt to reduce the number of children missed by vaccination and as a part of sentinel surveillance on National Immunization Days [21]. As a result, there were only eight cases of WPV in 2017, with a 97% reduction in polio cases since 2014 to 2017 [21].

However, progress against Polio in Pakistan has recently stagnated. Specifically, an ongoing challenge to further reduce vaccine derived polio virus in children and eliminate wild polio incidence is the shared polio reservoir and peripatetic movement of people between Pakistan and Afghanistan along the border area. Addressing this problem requires high level coordination and bilateral support between the two countries, strengthening Pakistan’s immunization programs, and engagement in concerted global health diplomacy (GHD) efforts [22].

### FATA: The nexus of polio

The FATA are conflict-affected areas on the border between Pakistan and Afghanistan that have seen a noticeable increase in the prevalence and incidence of wild polio cases compared to other parts of Pakistan. For security reasons, most FATAs ban civil interventions such as polio vaccination campaigns. These bans are designed to address terrorism activities in the area but can also relegate security threats at a higher local priority compared to public health-related prevention programs.

Beyond security concerns, the health sector in FATA is in disrepair. In Pakistan, inequalities exist in immunization coverage at the provisional level. For example, vaccination coverage is estimated at 75% in Punjab, but only 45% in FATA and Baluchistan [23]. Social inequality in this area is also related to the population’s lower education level, religious misconceptions, local cultural beliefs, and the long-conflicted border with Afghanistan [23].

The lack of safe drinking water in this area also contributes to the spread of fecal–oral infectious diseases like polio. Moreover, diarrheal diseases reduce absorption of polio vaccine and can reduce its efficacy indirectly [24]. Illustrating this risk, during border conflicts in 2015, 59 water treatment plants were destroyed in FATA [25]. Lack of safe drinking water, a gender gap, and low literacy rates collectively contribute to a rise in risks related to wild virus AFP associated polio cases [23]. This is further exacerbated by low infant immunization rates, leading to an increase in the number of new wild polio cases specific to this region.

Other social determinants of health also contribute to the risk of polio’s spread in FATA. Based on a survey conducted by the FATA Secretariat and the Bureau of Statistics, the gender gap in literacy and education is exceedingly wide: adult males are 45% literate, compared to as low as 7.8% among women (overall literacy rate of 28.4% in FATA compared to the 57% national average) [26]. Generally, people in the region are less educated due to violent conflict, where attacks in the territory destroyed an estimated 360 schools in 2015 [14]. These social-economic and educational inequities make it difficult for polio campaign staff to convey their health promotion messages [27].

Efforts to address these social determinants of health are ongoing. For example, improving parental health education is one of the milestones Pakistan must achieve to reduce the under-five mortality rate and positively impact polio eradication efforts. In a recent study of two-parent households, results indicated that the father’s decisions, especially in the rural communities, impacted vaccination coverage decisions in the family [28]. These findings indicate that improved education and targeted health promotion and interventions could improve vaccination coverage and prevention of other treatable diseases.

## Discussion

### Vaccine diplomacy

Global health diplomacy is a field having dual goals of improving health and strengthening relations among countries [29]. This form of “soft” or “smart” diplomacy is an essential tools in modern diplomatic practice, expanding contemporary areas of economic, political and military diplomacy to the health space [30]. Nestled within the broader practice of health diplomacy is “vaccine diplomacy” -- a branch of GHD that promotes the use and delivery of vaccines to achieve larger global health goals and shared foreign policy objectives [31]. Hence, promoting the use and availability of vaccines in low- and middle-income countries such as Pakistan operationalizes vaccine and health diplomacy, particularly important in the context of global polio eradication efforts.

Other examples of vaccine diplomacy involve addressing essential medicines access and affordability, where populations have been denied vaccines access due to intellectual property (IP) considerations, potentially endangering the global response to emerging infectious disease threats. This disagreement over IP led Indonesia to cease sharing its time-sensitive data about flu samples, which led to negotiation of the WHO Pandemic Influenza Preparedness Agreement, another pivotal case study in vaccine diplomacy [32].

Pakistani territories on the border of Afghanistan, particularly the FATA region and Baluchistan, have made it difficult to implement vaccine coverage, a situation that also represents a potential threat to neighboring countries’ health security and could reignite the international spread of polio. Hence, a more holistic approach employing the diplomatic tool of “vaccine diplomacy” to address Polio in Pakistan should be employed, which requires coordinating multiple areas of leadership, advocacy, policy, and global governance including around specific areas of:

1. Increasing national access to and ensuring the safety of the vaccine supply chain;
2. Improving water and sanitation in affected areas;
3. Enhancing environmental surveillance;
4. Mobilizing community and religious leaders;
5. Assisting Internally Displaced Persons (IDPs); and
6. Effective vaccine health promotion and communication activities

We provide more in-depth information regarding these specific recommendations, their primary challenges in the context of Polio eradication, and potential solutions that can be employed as summarized in Table 1. However, key to the success of achieving the above objectives are ensuring sustained funding and infrastructure for local, national, and global Polio eradication efforts, while at the same time achieving shared security and bilateral cooperation between Pakistan and Afghanistan.

**Table 1:** Summary of GHD Recommendations for Polio in Pakistan

Recommendation	Challenge(s)	Potential Solutions
*Increased access to vaccines*	*Supply Chain*: Frequent power outages in Pakistan, especially in rural areas, make it difficult to maintain the required temperature for proper polio vaccine transport and storage; polio vaccine requires refrigeration to maintain the cold chain, and improper temperatures can render vaccine ineffective. A vaccine campaign’s efficacy is hampered when ineffective and expired vaccines are given to populations, particularly those in hard to reach areas. If a cold chain breaks down at the domestic level, money, time, and lives are potentially at risk. *Access*: A fundamental priority is to increase access to free, effective, and easily administered polio vaccine to tackle remaining polio transmission avenues.	*Supply Chain*: Improving vaccine cold storage in tandem with enhanced regulatory control can improve vaccine efficacy. This includes use of random unscheduled inspection of health centers that store vaccine to monitor for compliance to cold chain storage that can further support the eradication process by identifying weaknesses in the vaccine supply chain. *Access*: Public health centers offer the oral polio vaccine free of cost and in August 2015, Pakistan started an injectable polio vaccine program with plans to vaccinate 6 million children^a^. Although the new injectable vaccine is more expensive than the OPV and requires a health care professional to administer, it can be more effective than OPV because only a single dose engenders immunity. Moreover, IPV has less chance to produce vaccine-derived polio virus and its related paralysis.
*Improving water and sanitation in affected areas*	FATA and other affected border areas are facing a serious water scarcity problem as the region lacks dams for water storage resulting in poor sanitary conditions. The combination of lack of literacy, false cultural beliefs and reduced water supply in border areas have resulted in reduced hand washing, even though Islam emphasizes the practice of frequent handwashing in the Quran to reduce disease risk. Tainted water and poor sanitary conditions transmit fecal–oral route infections like polio, hepatitis-A, and typhoid, and must be addressed in any comprehensive vaccine diplomacy efforts.	Access to clean drinking water must be prioritized as a fundamental human right and as a function of ensuring global health security and ending polio. Partner organizations such as Rotary International, UNICEF, RESULTS (an organization whose goal is to end poverty in the world), the UN Foundation, the Global Poverty Project, the World Bank and the Bill and Melinda Gates Foundation can help provide vulnerable populations with water treatment plants that ensure safe water supply. These efforts should integrate with Polio eradication efforts and should be specifically targeted for polio at-risk areas including border and rural communities.
*Enhancing environmental surveillance*	Environmental surveillance has detected polio virus in all provinces in Pakistan, although positive environmental samples decreased from 38% in 2014 to 16% in 2017.^b^ Laboratory tests on sewage indicate the presence of under-immunized or unimmunized children who can transmit the virus and contribute to its continuing spread.^c^ These children might have been immunized with expired and thus ineffective polio vaccines if the cold chain was not maintained during transport, while administering the vaccine, or if the children were missed cases.	To better quantify if these polio virus environmental trends are perpetuating, continued environmental surveillance of wastewater and sewage must be a priority. Positive environmental samples can serve as a proxy indicator for missed or unimmunized children that are not captured by other surveillance methods.
*Mobilizing community and religious leaders*	Mobilization of the community and religious leaders is important in health promotion efforts and achieving higher vaccine coverage. Religious leaders and the Imam Masjid (the worship leader of a mosque) can play a vital role in motivating people and improving community participation in polio eradication programs. The people of FATA and other affected border areas live in communities with strong religious beliefs and are oftentimes more conservative than Pakistani populations that live in other provinces. Additionally, community health workers need to be appropriately trained and incentivized.	*Religious Leaders*: The Imam and other religious leaders can help educate their communities that immunizing children and adults will eradicate polio. In this sense, religious leaders may be more persuasive and effective champions by practicing informal and grassroots vaccine diplomacy than national politicians or health staff due to the familiarity with culture and customs specific to affected areas of Pakistan that remain reservoirs for polio transmission.^d^ Fatwas (religious decisions) from Saudi Arabia in 2014, supporting polio vaccination and delivery of messages direct to the FATA and other border communities can also enhance community engagement, buy-in from religious leaders, and increase vaccination participation to ensure better compliance with polio eradication programs.^e^ *Community Health Workers*: Strengthening community health staff capacity through mandatory workshops on how to administer polio vaccines are also vital steps toward countrywide eradication. This includes ensuring there are appropriate incentives for health workers and a reliable monthly salary so that campaigns can be successfully carried out. Female staff availability in areas where women observe the hijab can also break down cultural barriers, support religious beliefs, and thereby enhance polio vaccination coverage. For example, a study using smart phone monitoring showed that increasing incentives for senior vaccinators could lead to increased vaccination coverage.^f^
*Assisting Internally Displaced Persons*	Movement of people between different regions internal to a country as a result of armed conflict, generalized violence or violation of human rights is termed “internal displacement of people.” Internally displaced people (IDP) are at particular risk for higher rates of unimmunized children, including in populations from south and north Waziristan, where conflict and displacement has created an immunity gap.^g^ The percentage of children missed due to polio teams not showing up is highest in FATA and Baluchistan.^g^ Pockets of unimmunized children are chronically missed because of internal migration and displacement in these conflicted areas.^g^ Internal displacement also has the potential to threaten broader disease transmission to larger cities like Karachi, Lahore, and Islamabad, when unimmunized IDPs are resettled and introduced into new communities.	Recognition of the human and health rights of IDPs and the unique health and security risks they face is important. This begins with clarification of their legal status and their right to access health services. IDPs should be prioritized in Polio eradication and vaccination interventions as an at-risk group. These groups should also be assessed for their risk of polio transmission in the context of forced migration or political displacement.
*Effective health promotion and communication*	Public service and mass media campaigns broadcast over radio can improve the regional population’s knowledge about benefits of polio vaccination. Announcements from religious leaders on both radio and digital channels such as YouTube can act as a powerful health promotion and vaccine awareness tool if utilized properly.	People in border areas communicate more frequently through mobile phones compared to traditional mass media forms such as television broadcast. In response, mobile applications using m-health (mobile Health) interventions have been deployed to help polio eradication teams increase their vaccination coverage in affected areas.^h^ Mobile phones are thus changing the way people access data and allowing public health interventions to expand to communities that may be remote or hard to engage with. In Pakistan in 2010, for example, mobile SMS-based service enabled parents to report missed cases of polio vaccine coverage.^h^

Sources

^a^ http://www.emro.who.int/pak/pakistan-infocus/introduces-ipv-in-routine-immunization.html

^b^ https://www.ncbi.nlm.nih.gov/pmc/articles/PMC5059991/

^c^ http://polioeradication.org/wp-content/uploads/2016/11/Inter-country-coordination-meeting-WPVCommonReservoirs_September2016.pdf

^d^ https://www.bbc.com/news/magazine-26734465

^e^ https://www.dawn.com/news/1167750

^f^ http://polioeradication.org/wp-content/uploads/2016/07/2.2_12IMB.pdf

^g^ https://www.ncbi.nlm.nih.gov/pmc/articles/PMC4750438/

### Funding and polio infrastructure

The Global Polio Eradication Initiative (GPEI) was launched in 1988, supervised by six organizations including the WHO, Rotary International, UNICEF, CDC, and the Bill and Melinda Gates Foundation. GPEI’s key strategy is to achieve polio eradication by immunizing every at-risk child until no child is left to transmit polio, effectively eliminating the reservoir of the disease and ending its transmission cycle. Reflecting the success of global efforts, the number of polio cases dropped more than 99% since 1988 as a result of coordinated initiation of polio eradication services in many countries [33].

According GPEI, global polio eradication funding efforts have experienced progress, but are still short an additional $1.5 billion [34]. Continuous investment in GPEI is economically viable because eradication can be more cost-effective than control through routine immunization. GPEI uses its funding to improve vaccine supply chain and update laboratory systems and social mobilization networks, all of which can help eradicate polio. Appropriate technology adoption and effective communication between national health centers’ clinical and administrative staff, as well as between patients, can help save both money and lives that is enabled by globally coordinated funding mechanisms deployed by GPEI.

Importantly, international partnerships such as GPEI represent a multilateral health diplomacy activity and also act as vehicles for resource mobilization directly to Pakistan. For example, Germany provides funding to Pakistan in the amount of an estimated 2 million Euros to eliminate the remaining reservoirs of polio virus [22]. Hence, continued support for GPEI and other international organizations engaged in the fight against global polio helps bolster domestic efforts by providing critical funding and building up national capacity needed to improve vaccine coverage and carry out prevention programs.

### Bilateral political commitment and border security

The border between Afghanistan and Pakistan is a sensitive area due to ongoing and long-term security threats complicated by the geography in the border belt area that is more mountainous and has a population that is sparsely distributed. This makes it challenging for health staff to reach and vaccinate every household in this region. The warlike conditions along the border aid in the persistent transmission of fecal–oral diseases in FATA and contiguous border areas. Syria exemplifies this problem: polio has gained a foothold due to conflict more than 14 years after the country’s successful eradication efforts, and now both Iraq and Lebanon are at risk of polio spread [35]. Reflecting this security risk, in Pakistan, around 36,000 children remained inaccessible to vaccination in North and South Waziristan following military operations in June 2014 at the border area between Pakistan and Afghanistan [9].

Stakeholder mechanisms to promote vaccine diplomacy in Pakistan and in the broader region include efforts to ensure inter-country coordination between Pakistan and Afghanistan. On September 21, 2016, the Common Reservoirs Coordination Meeting took place and included senior representatives from GPEI, WHO, UNICEF, the Bill and Melinda Gates Foundation, Rotary International, and the CDC, as well as senior representatives from each country [42]. The representatives came together to discuss disease outbreaks in the Pakistan and Afghanistan border areas, mainly in parts of Hangu (KP), Bannu, Dera Ismail Khan (KP), and areas bordering South Waziristan (Pakistan) and Paktika/Bermal (Afghanistan). Most outbreaks occurred in border regions between Pakistan and Afghanistan and eradication efforts emphasized the critical need for stable relationships between the governments to contain disease spread [36]. Health reporters stressed how improved bilateral notification and information reporting have enhanced polio surveillance, although issues surrounding delayed cross-border notifications still needed to be addressed [36].

As an example of opportunities for increased military engagement in polio operations, the Pakistan Armed Forces can help improve polio vaccination coverage by training soldiers in polio vaccination techniques to be used during military operations in affected areas. More direct vaccination enforcement may also be an option though is less desirable. For example, in 2015, the Khyber Pakhtunkhwa government issued arrest warrants to 1200 parents and guardians for their refusal to permit polio vaccine administration [37].

Hence, vaccine diplomacy activities need to be inclusive of both the multilateral environment, but also the national and regional military and security apparatus for affected countries. This is particularly important in the context of border and conflict areas that represent the final stronghold for polio.

## Conclusion

In November 2018, the WHO held its 19th meeting of the IHR Emergency Committee where it reviewed recent polio progress and also unanimously decided that the risk of international polio spread still constituted a PHEIC [38]. The committee again noted significant progress and cooperation by Pakistan and Afghanistan in reaching high-risk populations in border areas [38]. However, it also noted its concern regarding recent cross-border spread of wild poliovirus between the two countries, continued positive environmental isolates with wide geographic spread in Pakistan, and stagnation of the country’s eradication efforts (the number of polio cases was the same in 2017 and 2018) [38].

Hence, continued emergence of Polio indicates that Pakistan’s Polio Eradication Program requires continuous monitoring and surveillance in highly at-risk populations, policy action that is responsive to polio surveillance, and effective communication around the importance of polio vaccination as core interventions. Additionally, as we’ve outlined, there is a need for sustainable informative technology to deliver treatment, effective health communication and interventions, motivated religious leaders to engage with communities impacted, political commitment for funding and resource commitment, border stability to contain cross-border disease spread, and sustained global efforts to ensure adequate polio eradication funding.

Though Pakistan has faced setbacks in eradicating polio, analyzing these challenges through the lens of vaccine diplomacy can address key challenges at the local, national, and international level. Global policy recommendations generated through diplomatic negotiations and multistakeholder organizations (such as the WHO) can then elicit inter-country coordination with Pakistan’s immediate neighbors, and also enable resources for safe drinking water, and enhance public health under the broader lends of international development and health security. Complete eradication of polio through international and intersectoral coordination could then become a milestone achievement for shared goals in public health, international affairs, shared security, and diplomacy, and finally send Polio to the archives of history.

## Abbreviations

AFP: Acute Flaccid Paralysis
CDC: U.S. Centers for Disease Control and Prevention
CIA: U.S. Central Intelligence Agency
cVDPV: circulatory Vaccine Derived Polio Virus
DFID: United Kingdom Department for International Development
DG: Director General
FAO: Food and Agriculture Organization
FATA: Federally Administered Tribal Areas
GHD: Global Health Diplomacy
GPEI: Global Polio Eradication Initiative
IDP: Internally displaced people
IHR: International Health Regulations
IPV: Injectable Polio Virus
JICA: Japan International Cooperation agency
KPA: Khyber Pakhtunkhwa
NEAP: National Emergency Action Plan
OPV: Oral Polio Virus Vaccine
PHE: Public Health England
PHIC: Public Health Emergency of International Concern
USAID: United States Agency for International Development
WB: World Bank
WHO: World Health Organization
WPV: Wild Polio Virus
WPV1: Wild Polio Virus Type-1
